# Prevalence of Vitamin D Deficiency in Patients Admitted With Recent Fragility Hip Fracture: A Single-Center Retrospective Study

**DOI:** 10.7759/cureus.97239

**Published:** 2025-11-19

**Authors:** Chagigia Mousa, Christos Zafeiris, George Lyritis, Konstantinos Makris, Konstantinos Manolakos, Antonios Galanos, Efstathios Chronopoulos

**Affiliations:** 1 Orthopedic Surgery, Athens Medical Center, Athens, GRC; 2 Orthopedic and Spine Surgery, Metropolitan General Hospital, Athens, GRC; 3 Faculty of Medicine, Orthopedics, National and Kapodistrian University of Athens (NKUA), Athens, GRC; 4 Orthopedics, Faculty of Medicine, Hellenic Osteoporosis Foundation, Athens, GRC; 5 Clinical Biochemistry, KAT General Hospital, Athens, GRC; 6 Orthopedic Surgery, Laiko Hospital, Athens, GRC; 7 Epidemiology and Public Health, Laboratory for Research of the Musculoskeletal System, KAT General Hospital, Medical School, National and Kapodistrian University of Athens (NKUA), Athens, GRC; 8 Laboratory for Research on Musculoskeletal Disease, Orthopedic Surgery, National and Kapodistrian University of Athens (NKUA), Athens, GRC

**Keywords:** deficiency, elderly, hip fracture, new evidence, vitamin d

## Abstract

Hip fractures are the most common low‑energy injury affecting older adults and place a large burden on healthcare systems. Vitamin D sufficiency is fundamental to healthy bone turnover, maintenance of muscular strength, and immune system homeostasis. We aimed to evaluate the prevalence of vitamin D deficiency among patients admitted with recent fragility hip fractures at a single orthopedic clinic over one year. This retrospective study included patients aged >50 years admitted to the General Hospital of Athens “KAT” with fragility hip fractures during 12 months. Preoperative assessment included quantification of serum 25-hydroxyvitamin D (25(OH)D). Patients with bilateral hip fractures, chronic diseases affecting bone, polytrauma, and high‑energy trauma were excluded. Vitamin D status was categorized as deficiency <10 ng/mL, insufficiency 10-20 ng/mL, sufficiency >20 ng/mL. Of 186 patients presenting with hip fractures, 129 met the inclusion criteria (mean age 81.7 years; 72.9% female). Intertrochanteric fractures comprised 84 (65.1%). Deficiency of vitamin D was found in 72 (55.8%), insufficiency in 40 (31%), and sufficiency in 17 (13.2%). No statistically significant difference was observed between the sexes. Our data indicate a markedly high prevalence of vitamin D deficiency in older patients presenting with hip fractures. Consequently, we recommend introducing screening and prevention strategies.

## Introduction

Hip fractures are among the most consequential injuries in older adults, carrying substantial morbidity, mortality, and healthcare burden. As a key regulator, vitamin D maintains calcium homeostasis, supports skeletal mineralization, and facilitates neuromuscular activity. As a result, insufficiency/deficiency is widely documented at the population level across diverse regions and climates [1-6]. The prevalence is particularly high in older adults [7,8], extends to vulnerable groups such as pregnant women and neonates [9,10], and is frequently compounded by major comorbidities, including diabetes and obesity [11-13]. During the COVID-19 era, additional attention was drawn to systemic determinants of vitamin D status [14], while meteorological and seasonal effects further underscored geographic and temporal variability [15]. Beyond these epidemiologic patterns, inter-individual variability in vitamin D biology may be influenced by genetic differences within the vitamin D pathway, with several studies implicating vitamin D receptor (VDR) polymorphisms in skeletal outcomes and bone mineral density [16-19]. The present single-center, one-year study quantifies the prevalence of serum 25-hydroxyvitamin D (25(OH)D) deficiency among patients admitted with recent hip fractures, presenting current, locally relevant figures to shape peri-operative screening and supplementation, and to compare local practice with the published literature.

## Materials and methods

Patients 50 years of age and older with radiographically confirmed fragility hip fractures (including subcapital, transcervical, and basicervical femoral neck fractures, as well as intertrochanteric/subtrochanteric fractures) were eligible for this retrospective analysis, admitted through the Emergency Department to a single Orthopedic Clinic of the General Hospital of Athens “KAT” over 12 months. As part of the routine preoperative laboratory workup, serum vitamin D levels were quantified.

Patients aged <50 years, those with high-energy injuries or polytrauma, were excluded. In addition, patients with bilateral hip fractures; a medical history of conditions affecting bone density (e.g., advanced chronic kidney disease, chronic liver failure, malabsorption syndromes, primary hyperparathyroidism, uncontrolled hyperthyroidism, Cushing’s syndrome, or granulomatous disorders); those taking medications that alter vitamin D metabolism (e.g., enzyme-inducing antiepileptics, rifampicin) or long-term glucocorticoids (≥5 mg prednisolone-equivalent daily for ≥3 months); those with active malignancy; recent pharmacologic vitamin D therapy within the previous one to three months; or current anti-osteoporotic treatment were not included in the analysis (Table 1).

**Table 1:** Participants excluded before analysis.

Exclusion criteria	Patients
Age <50 years	1
High-energy injury mechanism or polytrauma	1
Simultaneous bilateral hip fracture	0
Chronic conditions affecting bone/vitamin D metabolism	2
Medications that alter vitamin D metabolism	4
Active malignancy	16
Recent pharmacologic vitamin D therapy	23
Current anti-osteoporotic drugs	8
Missing/delayed preoperative 25(OH)D measurement	2
	*n* = 57

**Figure 1:**
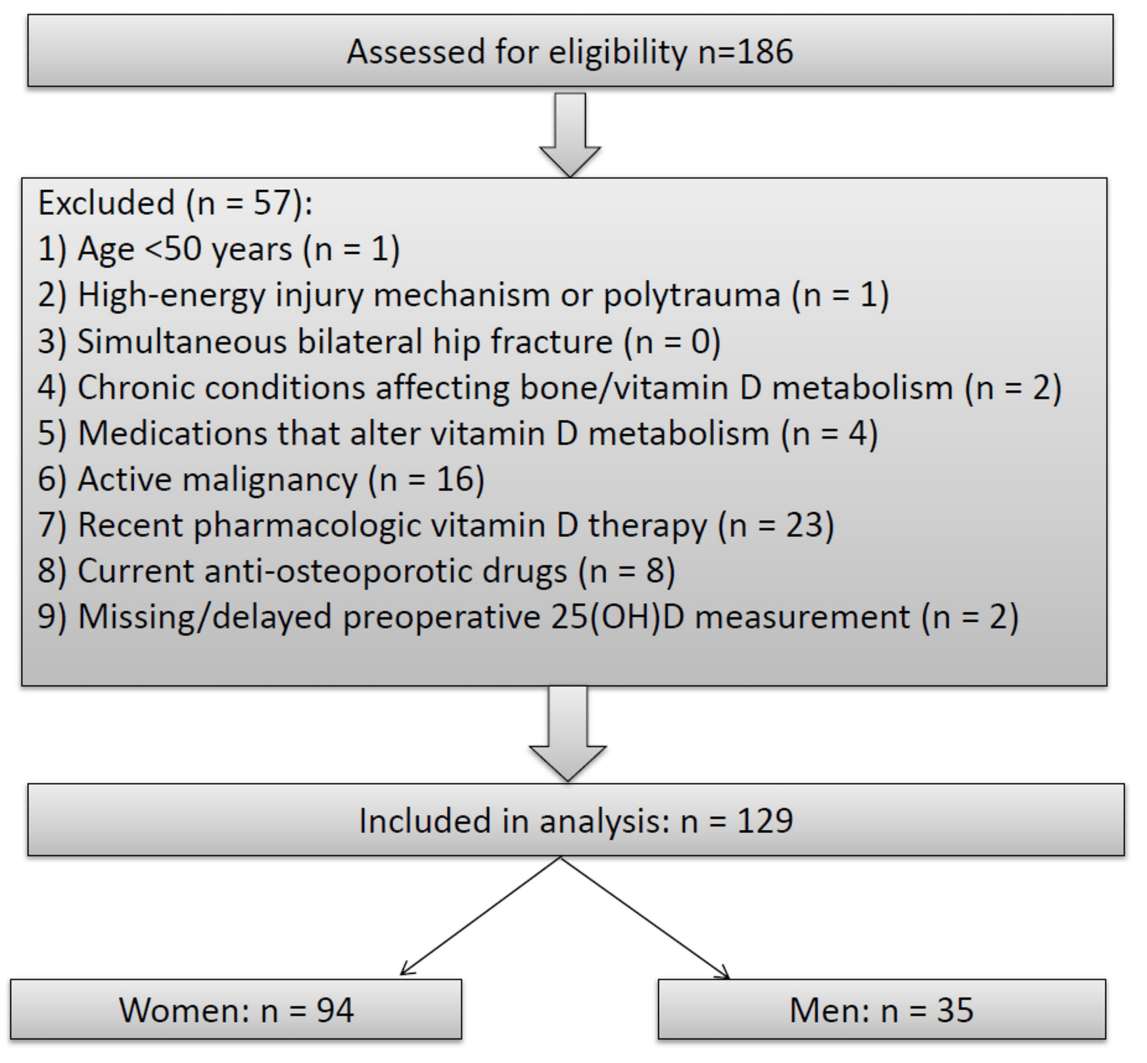
Study flow diagram: assessed (n = 186), excluded (n = 57), and included (n = 129). Image credit: Chagigia Mousa.

Of the 186 patients screened, 129 met eligibility criteria and were analyzed (Figure 1).

Fisher’s exact test was employed for statistical analysis as the appropriate method for assessing statistical significance in 2 × 2 contingency tables with categorical data. We conducted univariable logistic regression analyses for sex and fracture type to quantify their independent effects on vitamin D deficiency (odds ratios (ORs), 95% confidence intervals (CIs)). We used SPSS software (version 21.0; IBM Corp., Armonk, NY) for all analyses. Statistical tests were two-sided, and *P *< 0.05 denoted significance.

25(OH)D was measured with the use of Elecsys® Vitamin D total III assay (Roche Diagnostics, Mannheim, Germany). The assay is a competitive quantitative electrochemiluminescent assay for the determination of total 25(OH)D in serum and plasma in adults. This method has been standardized using internal calibrators that are traceable to the ID-LC-MS/MS 25(OH)D Reference Measurement Procedure [20,21]. The ID-LC-MS/MS is traceable to the National Institute of Standards and Technology Standard Reference Material 2972 [22]. The total imprecision (CVa%) of this assay in our laboratory is <6%.

## Results

A total of 186 patients were screened, of whom 129 met the inclusion criteria. The mean age was 81.7 ± 11.8 years; 94 females (72.9%) and 35 males (27.1%). Intertrochanteric fractures occurred in 84 patients (65.1%), and femoral neck fractures in 45 patients (34.9%). The left side was affected in 73 patients (56.6%) (Table 2).

**Table 2:** Distribution of the study population (%) by age, sex, fracture type, and affected limb.

	Total (*Ν *= 129)
Age (years)	Mean 81.7 ± 11.8
Women/men	94 (72.9%)/35 (27.1%)
Intertrochanteric/femoral neck fracture	84 (65.1%)/45 (34.9%)
Hip fracture: Right/left	56 (43.4%)/73 (56.6%)

**Figure 2:**
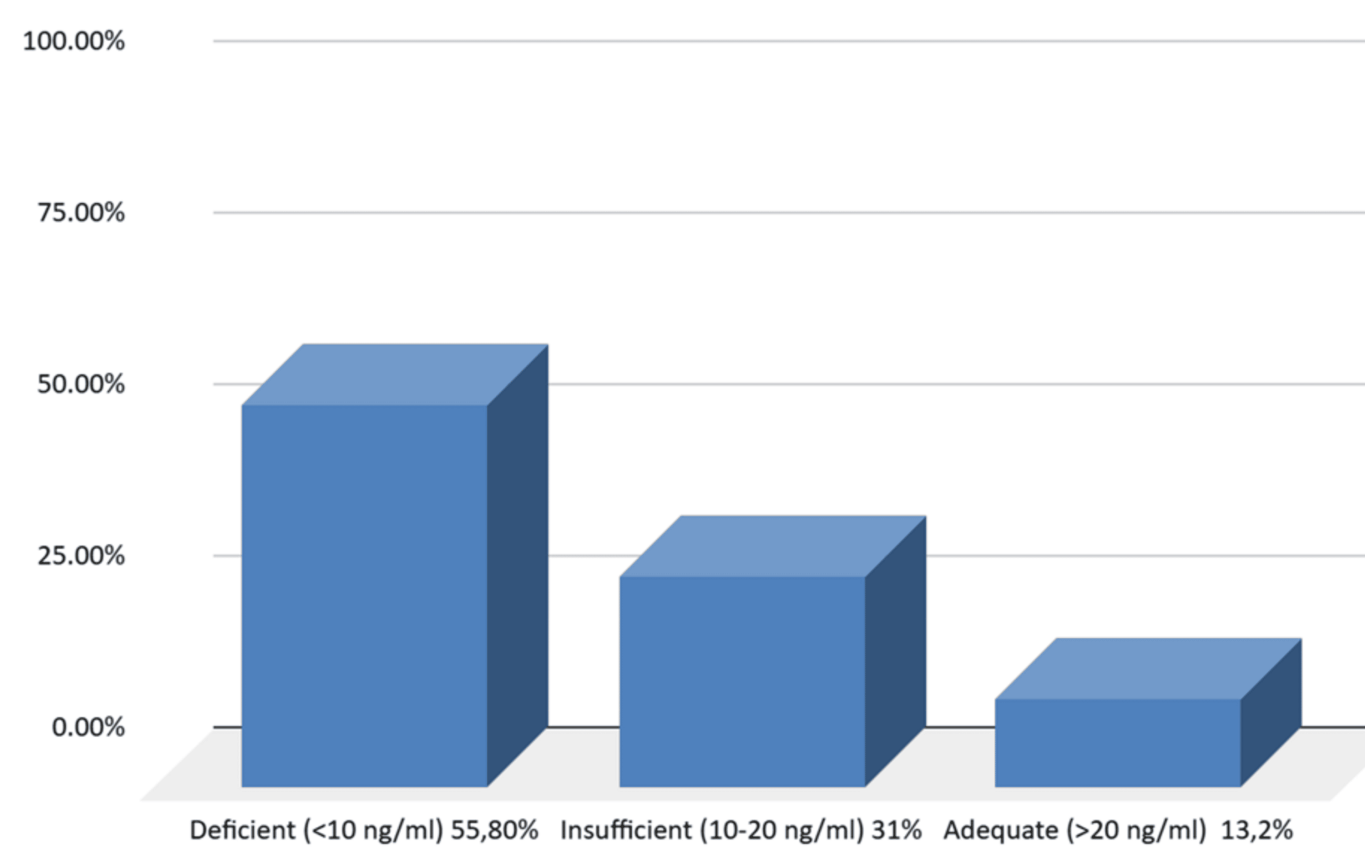
Proportion of patients with 25(OH)D deficiency among all admissions to an orthopedic clinic over a one-year period. 25(OH)D, 25-hydroxyvitamin D

We classified 25(OH)D levels in patients with hip fractures into three categories. Adequate vitamin D status was defined as levels >20 ng/mL. Levels between 10 and 20 ng/mL were considered insufficient, while levels <10 ng/mL were defined as deficient. According to our results, 72 out of 129 patients (55.8%) were vitamin D deficient, 40/129 (31%) were insufficient, and only 17/129 (13.2%) had adequate vitamin D levels (Figure 2).

**Figure 3:**
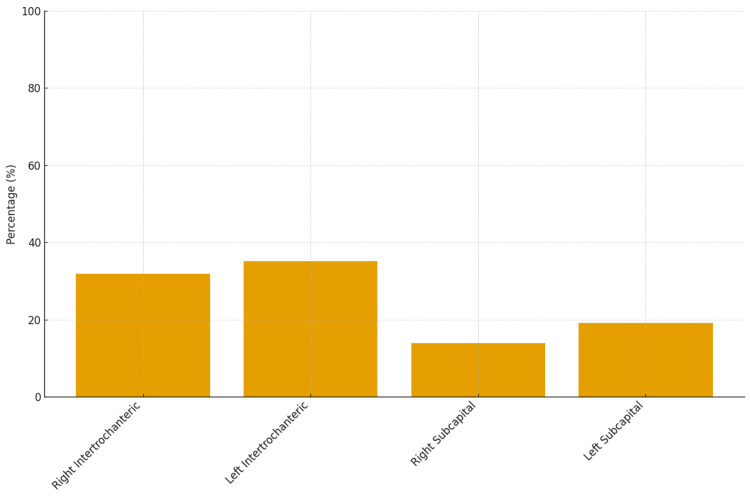
Distribution of hip fracture types among female patients admitted to an orthopedic clinic over a one-year period.

Among female patients, the mean age at the time of fracture was 83.2 ± 10.7 years. Trochanteric fractures accounted for 67% of cases, and 54.2% of fractures involved the left hip (Figure 3).

**Figure 4:**
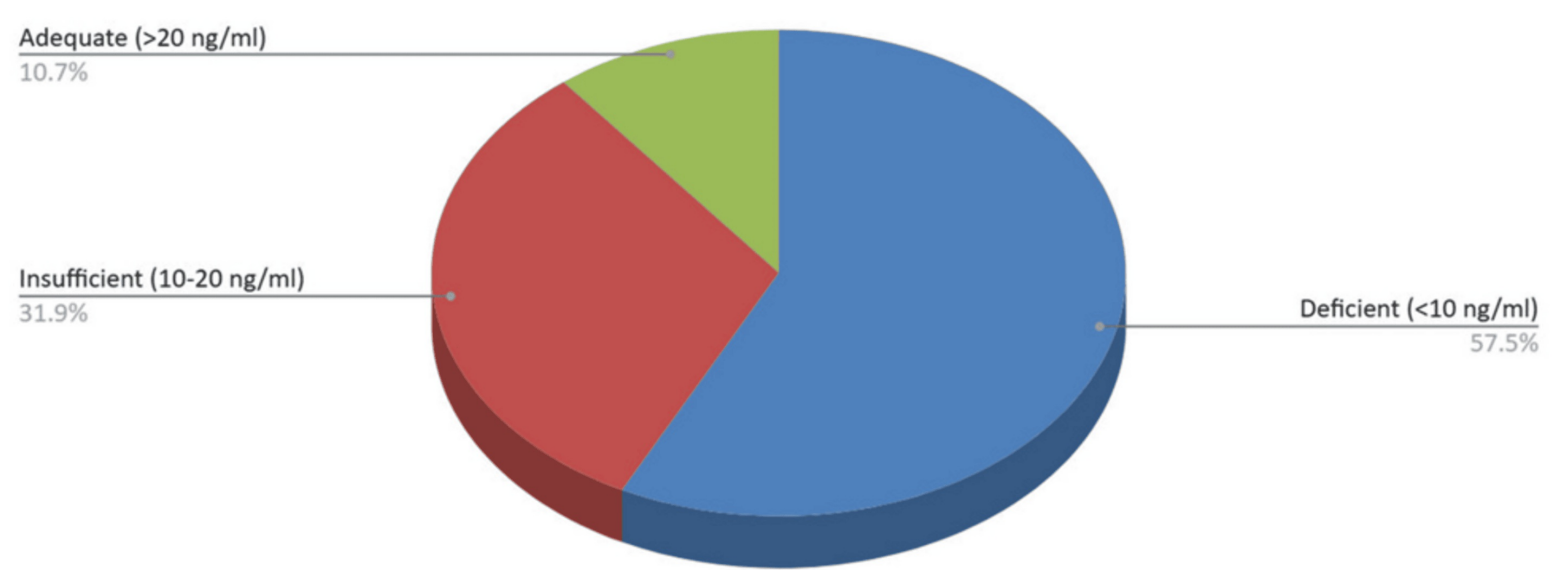
25(OH)D levels among female patients admitted to an orthopedic clinic over a one-year period. 25(OH)D, 25-hydroxyvitamin D

The prevalence of 25(OH)D deficiency in the female population was as follows: 54/94 (57.45%) were deficient, 30/94 (31.9%) were insufficient, and 10/94 (10.65%) had adequate vitamin D levels (Figure 4).

Among female patients with fractures, the mean 25(OH)D concentration was 12.1 ng/mL (SD 7.45). Postmenopausal women are the most vulnerable population for decreased bone mineral density due to hormonal changes. Our results reflect what has already been documented in the literature, showing an almost twofold increased risk for postmenopausal women to develop osteoporosis. Interestingly, the same injury mechanism led to trochanteric fractures in 67% of women, with no significant difference between the right and left sides. The overwhelming prevalence of vitamin D deficiency, with insufficiency and deficiency rates totaling approximately 89.35%, underscores the urgent need for increased awareness and proactive management of bone loss and early diagnosis by healthcare professionals. Fall prevention and the maintenance of bone health are crucial components that can influence and reduce the incidence of trochanteric fractures in the female population.

**Figure 5:**
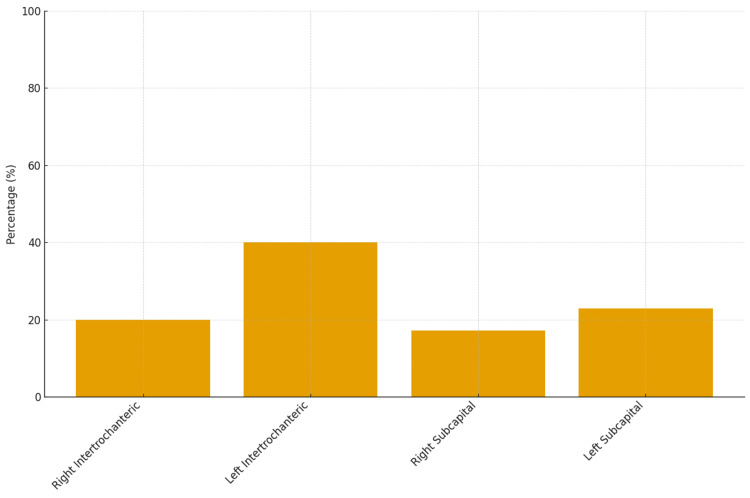
Distribution of hip fracture types among male patients admitted to an orthopedic clinic over a one-year period.

Regarding the male patients, the mean age at the time of fracture was 77.8 ± 13.2 years. Sixty percent of the fractures were intertrochanteric, and 63% involved the left hip (Figure 5).

**Figure 6:**
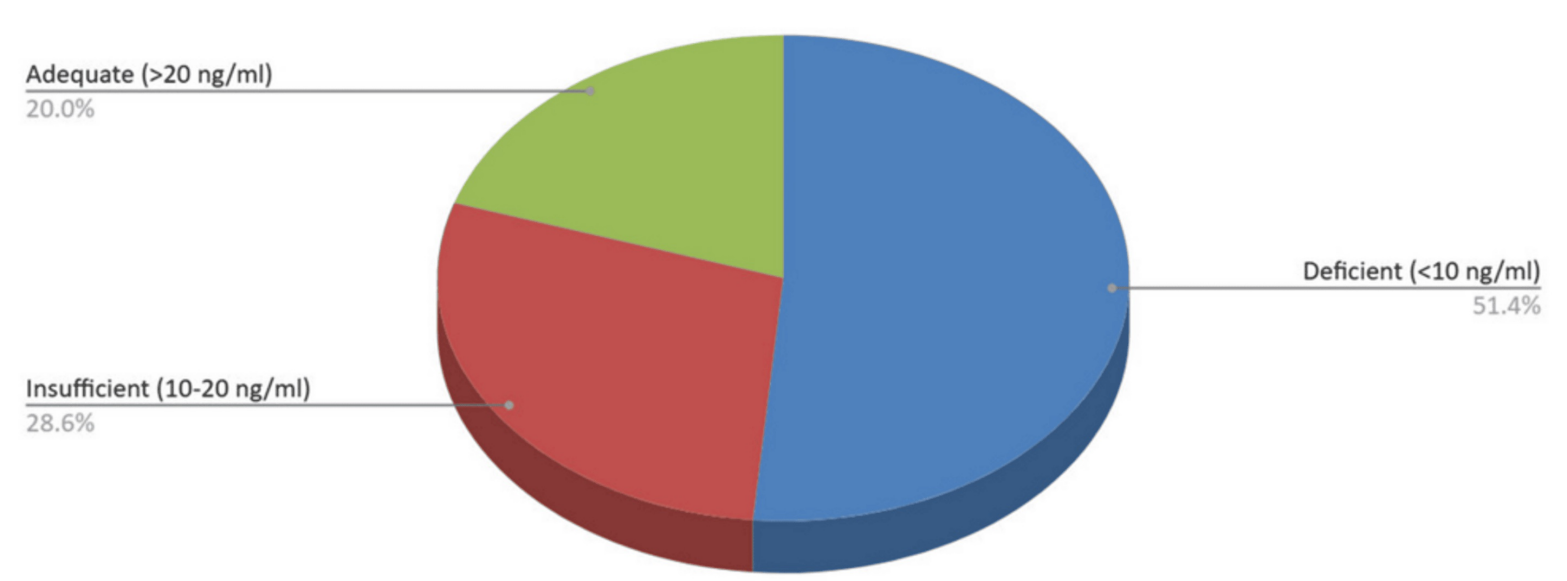
25(OH)D levels among male patients admitted to an orthopedic clinic over a one-year period. 25(OH)D, 25-hydroxyvitamin D

The prevalence of 25(OH)D deficiency among male patients was as follows: 18/35 (51.4%) were deficient, 10/35 (28.6%) were insufficient, and 7/35 (20%) had adequate vitamin D levels (Figure 6).

For male patients who sustained fractures, the mean 25(OH)D level was 14.2 ± 9.5 ng/mL. Although postmenopausal osteoporosis is a well-known phenomenon, the mean age of male patients at the time of fracture was lower than that of female patients. Trochanteric fractures predominated in this population as well, with a notable 63% involving the left hip. This may be related to the mechanism of injury and the fact that the dominant leg in most men is usually the right. Differences in the musculoskeletal system between men and women also likely contribute to balance and fall patterns. Overall, vitamin D insufficiency and deficiency remained high at approximately 80%, though still lower than that observed in women. These results highlight the need for increased awareness of osteoporosis in the male population.

Within this study women exhibited a higher prevalence of vitamin D deficiency than men, although the difference was not statistically significant (*P *= 0.239). In a logistic regression model, female sex was associated with 2.1-fold higher odds of deficiency (OR 2.1; 95% CI, 0.7-6.1; *P *= 0.169). Additionally, this logistic regression model indicated that individuals with intertrochanteric fractures had 2.4-fold higher odds of vitamin D deficiency compared with those with subcapital fractures (OR 2.4; 95% CI, 0.9-6.7; *P *= 0.100).

**Figure 7:**
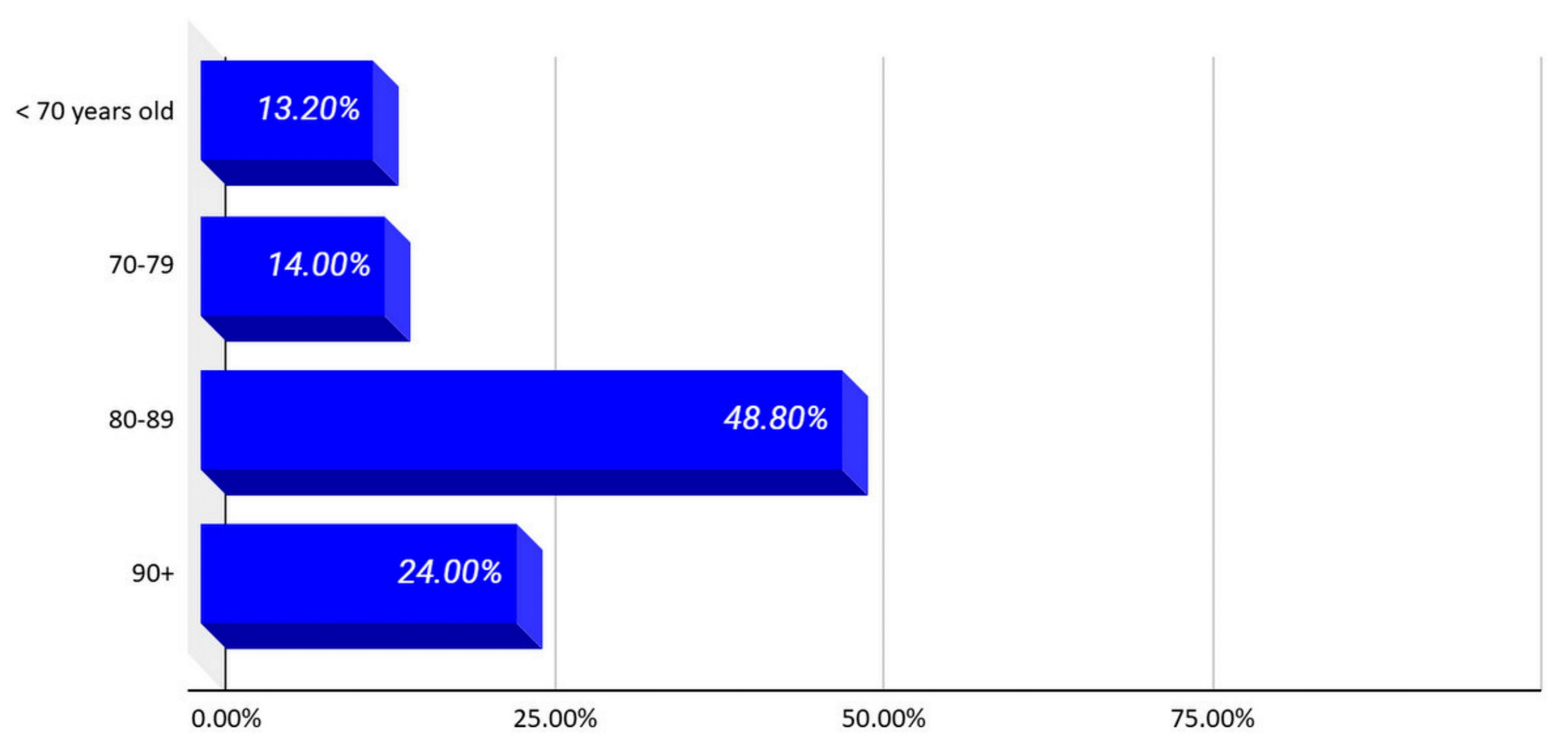
Distribution of study participants by age group.

As observed, the proportion of patients aged 70-79 years and those under 70 was nearly equal. Interestingly, patients aged 90 and above were not negligible, reflecting the increasing lifespan and the associated risk of falls and fractures in very elderly individuals (Figure 7).

Vitamin D levels across these age groups showed noteworthy patterns . Specifically, in the 70-90-year age range, vitamin D deficiency rates exceeded 90%, suggesting that this population should be a primary target for awareness and prevention programs (Table 3).

**Table 3:** Distribution of patients by vitamin D status.

			Adequacy-Insufficiency	Deficiency	Total
Age groups	<70 years old	Count	4	13	17
		%	23.5%	76.5%	100%
	70-79 years old	Count	0	18	18
		%	0%	100%	100%
	80-89 years old	Count	9	54	63
		%	14.3%	85.7%	100%
	90+ years old	Count	4	27	31
		%	12.9%	87.1%	100%
Total		Count	17	112	129
		%	13.2%	86.8%	100%

## Discussion

Our findings show a very high prevalence of low 25(OH)D at admission among patients with recent fragility hip fractures, consistent with international reports in comparable cohorts [23-27]. This corroboration strengthens the external validity of our observations while adding contemporary, region-specific estimates from a single Greek center. In line with population-based data demonstrating that latitude and sunshine alone do not ensure sufficiency [1-6,15], older adults in particular remain vulnerable due to reduced cutaneous synthesis, dietary inadequacy, multimorbidity/polypharmacy, limited outdoor activity, and seasonality [7,8,15].

Given vitamin D’s skeletal and extraskeletal actions, our results support routine perioperative assessment and correction. A pragmatic, auditable pathway would include: (1) universal 25(OH)D testing within 24 hours of admission for hip fracture; (2) immediate, guideline-concordant supplementation when deficiency/insufficiency is detected; (3) integration with fracture liaison services for coordinated osteoporosis work-up and fall-prevention; and (4) standardized discharge education with clear maintenance plans. To ensure fidelity, programs should embed electronic order sets and track process/outcome metrics (e.g., proportion screened and treated before discharge, adherence at 6-12 weeks, rehospitalizations, and refracture) [7,8,15,23-27].

Beyond the index admission, our data align with broader evidence that hypovitaminosis D is widespread across regions and patient groups [1-6,9-13,15]. We therefore emphasize sustained prevention, particularly for adults ≥65 years and other high-risk groups, through: periodic re-testing in winter/early spring; medication review (e.g., enzyme-inducing antiepileptics, rifampicin, chronic glucocorticoids); nutrition counseling and supplementation adherence checks; fall-prevention programs; primary-care recall systems for hip-fracture survivors; and harmonized laboratory thresholds and sampling timing to improve comparability across sites [7,8,11-13,15]. These steps situate local practice within the existing literature and translate our in-hospital findings into durable, population-level benefit.

In interpreting these findings, several considerations merit mention: the single-center, retrospective design, a relatively modest sample size, and the absence of seasonal vitamin D information, as well as potential residual confounding (e.g., comorbidity burden, functional status) and between-assay/threshold variability, all of which may temper cross-study comparisons [3,7,8,15,16-19,23-27]. Even so, the consistently high prevalence of low 25(OH)D supports pragmatic changes to care: we recommend routine screening at admission, particularly for adults ≥65 years, prompt, guideline-concordant supplementation when deficiency/insufficiency is detected, and standardized follow-up (e.g., re-test at 6-12 weeks), supported by electronic order sets and clear links to fracture liaison services. Multicenter prospective studies-and, where feasible, randomized trials-should further evaluate whether rapid correction improves clinical outcomes and refine dosing and adherence strategies, including the potential influence of genetic modifiers [3,7,8,15,16-19,23-27].

## Conclusions

We found a markedly high prevalence of low 25(OH)D at admission among older adults with recent fragility hip fractures treated at our center over one year, across sexes, age groups, and fracture types-indicating a modifiable gap in routine care and a broader public-health concern. We, therefore, recommend routine preoperative 25(OH)D screening within 24 hours of admission, prompt guideline-concordant supplementation for deficiency/insufficiency, and integration with fracture liaison services to align vitamin D correction with osteoporosis treatment and fall-prevention. Programs should also track implementation metrics (e.g., proportion screened and treated before discharge, early adherence).

At the population level, targeted measures aligned with national guidance to improve vitamin D status in older adults are warranted. Multicenter prospective studies and randomized trials should evaluate whether rapid correction around the time of fracture improves short-term outcomes (complications, length of stay, early mortality) and long-term endpoints (refracture, functional independence), while clarifying dose-response, adherence, seasonal effects, comorbidity interactions, and potential genetic modifiers (e.g., VDR variants) to identify who benefits most and to support pragmatic, scalable protocols.
